# Surveillance for highly pathogenic influenza A viruses in California during 2014–2015 provides insights into viral evolutionary pathways and the spatiotemporal extent of viruses in the Pacific Americas Flyway

**DOI:** 10.1038/emi.2017.66

**Published:** 2017-09-06

**Authors:** Andrew M Ramey, Nichola J Hill, Troy Cline, Magdalena Plancarte, Susan De La Cruz, Michael L Casazza, Joshua T Ackerman, Joseph P Fleskes, T Winston Vickers, Andrew B Reeves, Frances Gulland, Christine Fontaine, Diann J Prosser, Jonathan A Runstadler, Walter M Boyce

**Affiliations:** 1 US Geological Survey, Alaska Science Center, 4210 University Drive, Anchorage, AK 99508, USA; 2 Massachusetts Institute of Technology, Cambridge, MA 02139, USA; 3 Department of Biological Sciences, California State University, Chico, CA 95929, USA; 4 School of Veterinary Medicine, Wildlife Health Center, University of California Davis, One Shields Avenue, Davis, CA 95616, USA; 5 US Geological Survey, Western Ecological Research Center, San Francisco Bay Estuary Field Station, 505 Azuar Drive, Vallejo, CA 94592, USA; 6 US Geological Survey, Western Ecological Research Center, Dixon Field Station, 800 Business Park Drive, Suite D, Dixon, CA 95620, USA; 7 The Marine Mammal Center, 2000 Bunker Road, Sausalito, CA 94965, USA; 8 US Geological Survey, Patuxent Wildlife Research Center, Beltsville Lab, 10300 Baltimore Avenue, Beltsville, MD 20705, USA

**Keywords:** California, clade 2.3.4.4, H5, highly pathogenic, influenza A, pinniped, virus, waterfowl

## Abstract

We used surveillance data collected in California before, concurrent with, and subsequent to an outbreak of highly pathogenic (HP) clade 2.3.4.4 influenza A viruses (IAVs) in 2014–2015 to (i) evaluate IAV prevalence in waterfowl, (ii) assess the evidence for spill-over infections in marine mammals and (iii) genetically characterize low-pathogenic (LP) and HP IAVs to refine inference on the spatiotemporal extent of HP genome constellations and to evaluate possible evolutionary pathways. We screened samples from 1496 waterfowl and 1142 marine mammals collected from April 2014 to August 2015 and detected IAV RNA in 159 samples collected from birds (*n*=157) and pinnipeds (*n*=2). HP IAV RNA was identified in three samples originating from American wigeon (*Anas americana*). Genetic sequence data were generated for a clade 2.3.4.4 HP IAV-positive diagnostic sample and 57 LP IAV isolates. Phylogenetic analyses revealed that the HP IAV was a reassortant H5N8 virus with gene segments closely related to LP IAVs detected in mallards (*Anas platyrhynchos*) sampled in California and other IAVs detected in wild birds sampled within the Pacific Americas Flyway. In addition, our analysis provided support for common ancestry between LP IAVs recovered from waterfowl sampled in California and gene segments of reassortant HP H5N1 IAVs detected in British Columbia, Canada and Washington, USA. Our investigation provides evidence that waterfowl are likely to have played a role in the evolution of reassortant HP IAVs in the Pacific Americas Flyway during 2014–2015, whereas we did not find support for spill-over infections in potential pinniped hosts.

## INTRODUCTION

Highly pathogenic (HP) H5 influenza A viruses (IAVs) of the Goose Guangdong lineage were first detected in China in 1996.^1^ During the subsequent two decades, viral descendants that share ancestry at the hemagglutinin gene segment have evolved through reassortment with viruses of numerous neuraminidase (NA) subtypes, resulting in the emergence and intercontinental spread of novel HP IAVs designated as belonging to clade 2.3.4.4.^2, 3, 4, 5, 6, 7, 8, 9, 10^ In autumn 2014, Goose Guangdong lineage clade 2.3.4.4 HP IAVs were detected in wild, captive and domestic birds in North America.^5, 6, 11^ Purportedly, clade 2.3.4.4 HP H5N8 IAVs were introduced into North America via migratory waterfowl.^11^ Although definitive proof for this hypothesis remains elusive, this proposed mechanism of introduction is consistent with (i) intercontinental migratory movements of potential waterfowl hosts,^12, 13, 14^ (ii) previous evidence for viral dispersal between East Asia and North America by wild birds,^15^ (iii) the widespread detection of HP H5N8 and descendant viruses in wild birds in the Pacific Americas Flyway during 2014–2015,^16^ (iv) a lack of pathobiological effects or barriers to infection in experimentally inoculated waterfowl^17^ and (v) the circulation of low-pathogenic (LP) viruses in wild waterfowl sampled in Alaska that share recent common ancestry with reassortant viruses descended from Eurasian lineage HP H5N8.^18, 19^ Therefore, it is likely that waterfowl contributed to the introduction and dissemination of clade 2.3.4.4 HP IAVs in North America.

While surveillance for IAVs in waterfowl sampled in the Pacific Flyway has provided information on the prevalence and evolution of HP clade 2.3.4.4 viruses during the 2014–2015 outbreak,^16, 18, 19^ many aspects regarding the maintenance, reassortment and dispersal of HP clade 2.3.4.4 IAVs in North America remain poorly understood. For example, despite extensive active and passive surveillance efforts to detect clade 2.3.4.4 HP IAVs throughout the Pacific Flyway of North America during December 2014–January 2015,^16^ there is limited published information on the sampling of wild waterfowl in this region either prior or subsequent to the 2014–2015 outbreak, which may be useful for defining the spatiotemporal extent of this event. In addition, previously reported surveillance efforts for clade 2.3.4.4 HP IAVs targeted waterfowl and poultry, and thus, there is no published information with which to assess spill-over into potential pinniped hosts. While clade 2.3.4.4 IAVs have not previously been reported in marine mammals, prior investigations have identified IAVs and antibodies thereto in both free-ranging pinnipeds and those that stranded at coastal locations in North America.^20, 21, 22, 23, 24, 25^ Past association of avian-origin IAVs with pinnipeds that stranded and were found dead or moribund suggests that the effects of infection may not be negligible.^20, 21, 22^ Given the apparent widespread geographic distribution of clade 2.3.4.4 IAVs along the Pacific Coast of North America during 2014–2015, an assessment of infection in pinnipeds in outbreak-affected regions may be warranted. Finally, there has been only limited genetic characterization of LP IAVs from wild waterfowl sampled during 2014–2015 in the Pacific Americas Flyway outside of Alaska, which has resulted in information gaps regarding the evolutionary pathways of reassortant clade 2.3.4.4 HP IAVs in North America following the introduction of intercontinental group A (icA) HP H5N8.^11^

In this study, we explore the prevalence, evolution and spread of HP clade 2.3.4.4 IAVs in the Pacific Americas Flyway by using surveillance data collected in California before, concurrent with and subsequent to the previously reported outbreak of 2014–2015. Specifically, we evaluate the prevalence of HP clade 2.3.4.4 IAVs in waterfowl relative to previous reports and assess the evidence for spill-over infections in pinnipeds. Furthermore, we genetically characterize both LP and HP IAVs recovered through our sampling effort to refine inference on the spatiotemporal extent of HP clade 2.3.4.4 genome constellations in California during the outbreak and to evaluate possible evolutionary pathways. Our results supplement previous summaries of surveillance efforts for clade 2.3.4.4 HP IAVs in North America and extend inference relative to viral evolution. This information may be useful to better understand the role of wildlife in the maintenance and evolution of clade 2.3.4.4 IAVs in North America. Furthermore, our results may inform surveillance strategies and biosecurity recommendations for regulatory agencies, poultry producers and other stakeholders that are concerned with HP IAVs in the United States of America.

## MATERIALS AND METHODS

### Ethics statement

Sampling procedures that involved live animals as reported in this study were reviewed by the Institutional Animal Care and Use committees at the University of California (UC), the California State University and the US Geological Survey (USGS) (the UC permits No. 18034 and No. 19559; the California State University permit No. 2014-2; and the USGS permit WERC-2015-01), and were conducted under authorizations granted by the following permits: US Fish and Wildlife Service Special Use permit No. 81620-14-009; US Fish and Wildlife Service Scientific Collection permit No. MB191637-0; USGS Federal Bird Banding Permits No. 21142 and No. 22911; California Department of Fish and Wildlife Scientific Collection Permits No. 3855, No. 5000 and No. 8090; and National Oceanic and Atmospheric Administration National Marine Fisheries Service Marine Mammal Protection Act permit No. 18786-01.

### Sampling

**Figure 1:**
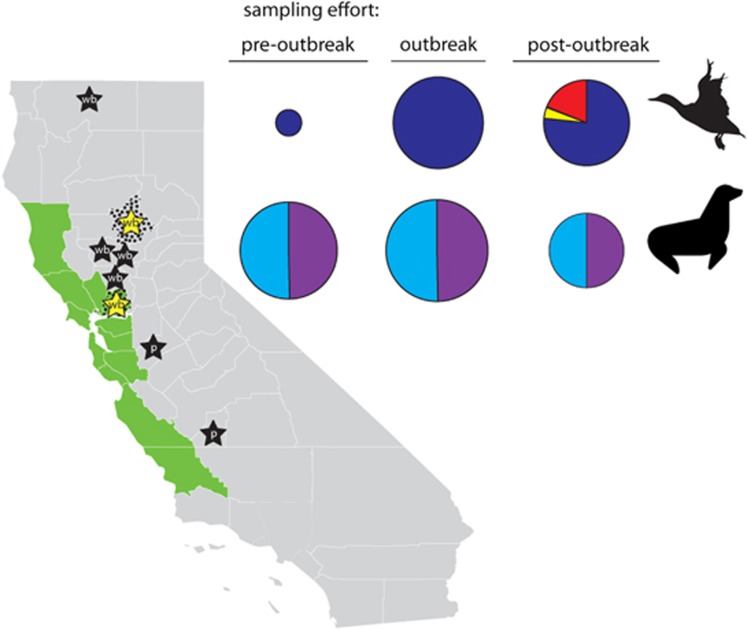
Map of California depicting locations at which surveillance samples were collected as part of this study. Butte and Solano counties, where samples were collected from wild waterfowl, are indicated with black stippling. The Central California Coast (Mendocino, Sonoma, Marin, Napa, Solano, Contra Costa, Alameda, Santa Clara, San Mateo, San Francisco, Santa Cruz, Monterey and San Luis Obispo counties), where pinnipeds stranded and where samples were collected, is depicted in green. Stars represent approximate county-level locations, where highly pathogenic clade 2.3.4.4 influenza A viruses have been previously detected in California in wild birds (wb: Butte; Colusa; Siskiyou; Solano; Sutter; and Yolo counties) and in poultry (p: Kings; and Stanislaus counties). Yellow stars depict the detection of highly pathogenic clade 2.3.4.4 influenza A viruses from samples that were collected from wild waterfowl sampled in Butte and Solano counties as part of the current study. Pie charts on the right-hand side depict the relative sampling effort (size of pie chart) and proportion of each sample type (cloacal swab=dark blue; fecal swab=yellow; oropharyngeal swab=black; paired oropharyngeal/cloacal swab=red; nasal swab=light blue; and rectal swab=purple) that originated from wild waterfowl and pinnipeds during pre-outbreak (April–August 2014), outbreak (September 2014–March 2015) and post-outbreak (April–August 2015) periods. Map of California courtesy of FreeVectorMaps.com (https://freevectormaps.com/united-states/california/US-CA-EPS-01-0002).

Cloacal swabs, oropharyngeal swabs and/or fecal samples were collected from live waterfowl in Solano County, California, from July through August in both 2014 and 2015, and from hunter-harvested waterfowl in Solano and Butte counties, California, from October 2014 through January 2015 (Figure 1). Live birds were captured in baited swim-in traps or by using rocket nets or were sampled by swabbing freshly deposited feces. Hunter-harvested waterfowl samples were collected at check stations. Swabs were collected using sterile polyester-tipped applicators, placed in vials containing 2 mL of cold virus transport medium (VTM: Medium 199 with Earle’s salts, L-glutamine and sodium bicarbonate plus 2 mU/L penicillin G, 200 mg/L streptomycin, 2 mU/L polymyxin B, 250 mg/L gentamicin, 0.5 mU/L nystatin, 60 mg/L ofloxacin, 200 mg/L sulfamethoxazole and 0.5% bovine serum albumin), and transported on ice to the laboratory where they were stored at −80 °C on the day of collection until processing. Swabs were placed individually in vials with VTM except for 129 samples from live-sampled birds, which contained pooled cloacal and oropharyngeal swabs.

From April 2014 to August 2015, pinnipeds that stranded along the Central Coast of California, as identified through the California Marine Mammal Stranding Network, and that were hospitalized for rehabilitation at The Marine Mammal Center in Sausalito, California, were sampled for IAVs as part of routine health examinations upon entry to the rehabilitation facility (Figure 1). Species sampled included California sea lions (*Zalophus californianus*), Guadalupe fur seals (*Arctocephalus townsendi*), northern elephant seals (*Mirounga angustirostris*), northern fur seals (*Callorhinus ursinus*), Pacific harbor seals (*Phoca vitulina*) and Steller sea lions (*Eumetopias jubatus*). Nasal and rectal swabs were collected from each animal, and the swabs were placed individually in vials containing VTM. Samples were refrigerated up to 1 week before shipping to UC Davis. Upon receipt at the UC Davis laboratory, samples were either further processed immediately or stored at −80 °C.

### Screening for IAVs

Viral RNA was extracted from all swab samples using MagMAX-96 AI/ND Viral RNA Isolation Kits (Ambion/Applied Biosystems, Foster City, CA, USA). RNA was screened using a two-step real-time reverse transcriptase-PCR (rRT-PCR) that targeted the IAV matrix gene.^26^ Any sample that did not yield a cycle threshold (Ct) value ≤45 was considered negative for IAV. Matrix gene rRT-PCR-positive samples were further screened using a HP clade 2.3.4.4 H5-specific rRT-PCR that utilized the following primers and probe: H5 EA+1462 (5′-TGG GTA ATG GTT GTT TCG AG-3′); H5 EA-1565 (5′-ATT GCT TCT TCT GAA TAC TTA GGG-3′); and H5EA 1487 probe ((6-FAM) 5′-TCA CAA ATG/ZEN/TGA TAA CGA ATG TAT GGA GAG C-3′ (IBFQ). The AgPath ID RT-PCR kit (Life Technologies, Carlsbad, CA, USA) and the AB 7500 Fast instrument (Life Technologies) were used to screen for HP clade 2.3.4.4 H5 IAV by applying the following thermocycling protocol: 45 °C for 10 min; 95 °C for 10 min; and then 40 cycles of 94 °C for 10 s, 57 °C for 10 s and 72 °C for 5 s. VTM samples that were identified as putatively positive for HP clade 2.3.4.4 H5 IAVs were sent to the California Health and Food Animal Safety Laboratory and to the US Department of Agriculture National Veterinary Services Laboratory for further testing. Virus isolation was attempted on all matrix-positive, HP H5 rRT-PCR-negative samples by inoculating 200 μL of VTM into the allantoic cavities of two 11-day-old embryonated chicken eggs (Charles River, North Franklin, CT, USA) and incubating at 37.8 °C for 3 days or until embryo death. Allantoic fluid from each pair of eggs was collected and pooled, and 50 μL of this fluid was processed for RNA extraction and rRT-PCR testing for the IAV matrix gene target as described above. Virus isolates were defined as any sample that yielded allantoic fluid with a Ct value ≤45 for the IAV matrix gene after being cultured in specific pathogen-free eggs.

### Sequencing of virus isolates

Full-genome sequencing was attempted on RNA that was extracted from all virus isolates at either UC Davis, the Icahn School of Medicine at Mount Sinai (ISMMS) or the USGS Alaska Science Center. For genomic sequencing at UC Davis and ISMMS, sequencing was attempted on RNA that was extracted from 55 isolates by first amplifying genomic RNA in a multisegment RT-PCR reaction by using 5 μL of RNA template, SuperScript III high-fidelity RT-PCR kit reagents (Invitrogen, Carlsbad, CA, USA) and influenza-specific universal primers (Opti1-F1-5′-GTT ACG CGC CAG CAA AAG CAG G-3′ at 0.1 μM, Opti1-F2-5′-GTT ACG CGC CAG CGA AAG CAG G-3′ at 0.1 μM and Opti1-R1-5′-GTT ACG CGC CAG TAG AAA CAA GG-3′ at 0.2 μM). After thermocycling, 5 μL of the product was visualized on a 0.8% agarose gel to verify the amplification of all genomic segments. Samples for which product for all eight gene segments was visually verified were purified with 0.45 × volume AMPure XP beads (Beckman Coulter, Brea, CA, USA) and sequenced at ISMMS by using methods reported by Mena *et al.*^27^ The GenBank accession numbers for the 27 complete IAV genomes that were sequenced at UC Davis and ISMMS as part of this study are KX351517–KX351766.

At the USGS Alaska Science Center, genomic sequencing was attempted on RNA extracts that were provided by UC Davis for 31 viral isolates and three diagnostic original swab samples for which Ct values ≤45 were obtained by rRT-PCR screening for HP clade 2.3.4.4 H5 IAVs. For each isolate, 2 μL of RNA template was amplified using SuperScript III One-Step RT-PCR System with Platinum Taq High Fidelity (Invitrogen) and the following primers: MBTuni-12 5′-ACG CGT GAT CAG CAA AAG CAG G-3′ at 0.1 μM, MBTuni-12(M) 5′-ACG CGT GAT CAG CRA AAG CAG G-3′ at 0.1 μM and MBTuni-13 5′-ACG CGT GAT CAG TAG AAA CAA GG-3′ at 0.2 μM. Thermocycling conditions were 42 °C for 60 min; 5 cycles of 94 °C for 15 s, 45 °C for 30 s and 68 °C for 3 min; and 30 cycles of 94 °C for 15 s, 57 °C for 30 s and 68 °C for 3 min. Following product visualization and verification (5 μL on a 1.0% agarose gel), excess deoxynucleotide triphosphates and primers were removed using ExoSAP-IT (USB Corporation, Cleveland, OH, USA). PCR products were quantified using a Quant-iT dsDNA HS Assay Kit (Invitrogen) and prepared for sequencing using a Nextera XT DNA library preparation kit (Illumina, Inc., San Diego, CA, USA). Indexed libraries were pooled and sequenced on an Illumina MiSeq using a 500-cycle reagent kit with paired-end reads. Sequence reads were assembled with Geneious 9.1.3^28^ using reference data for IAVs that were obtained from GenBank^29^ to map reads. Consensus sequences were verified using FLuANotation. The GenBank accession numbers for 28 complete and 3 partial IAV genomes that were sequenced at the USGS Alaska Science Center as part of this study are KY828600–KY828841.

### Phylogenetic analyses

IAV sequences that originated from samples collected in East Asia (Japan, South Korea and Taiwan) and North America (Canada and United States) from 2010 to 2016 were downloaded from the National Center for Biotechnology Information Influenza Virus Resource database in December 2016. Only full-length or near-full-length genomic segments that were associated with a full date of collection (DD/MM/YYYY) were included. Sequences for IAVs that were generated as part of this study were aligned with reference sequences using MUSCLE v3.8.31,^30^ inspected visually in Geneious v7.1.5 (North America Biomatters Inc., Newark, NJ, USA) and trimmed to remove nucleotides that were outside the coding region.

Maximum-likelihood trees using the GTRGAMMA substitution model in RAxML v8.1.16^31^ were reconstructed for each segment by using publicly available sequences and the sequences that were generated for this study, as described above (sequences per segment: PB2=1749; PB1=1806; PA=1802; H5=372; NP=1735; N1=248; N2=335; N8=602; M=1719; and NS=1760). Trees were visualized in FigTree v1.4.3 (http://tree.bio.ed.ac.uk/software/figtree/) and randomly down-sampled to reduce the number of sequences for Bayesian molecular clock phylogenetic analysis (~300 taxa). The down-sampling strategy preserved all clade 2.3.4.4 isolates from East Asia and North America, and concurrently circulating LP isolates from the Pacific Americas Flyway that originated from samples collected from 2014 to 2016 (PB2=345, PB1=353, PA=340, H5=338, NP=366, N1=248, N2=335, N8=306, M=362 and NS=370).

Bayesian phylogenetic trees for each segment were reconstructed using BEAST v1.8.3.^32, 33^ At least four independent markov chain Monte Carlo chains of 40–80 million generations each were run using a GMRF Bayesian skyride coalescent tree prior and uncorrelated lognormal distribution to produce 10 000 trees. To ensure that markov chain Monte Carlo chains converged on the same optimal tree, runs were visually inspected in Tracer v1.6.0 (http://tree.bio.ed.ac.uk/software/tracer/). After removing 10%–30% burn-in from each chain, the trees were combined to produce a single maximum clade credibility tree per genomic segment.

### Molecular dating analysis

The time of most recent common ancestry (tMRCA) and 95% highest posterior densities (95% HPDs) for clades of interest (see Results) was inferred from the nodes of the maximum clade credibility tree as inspected in FigTree v1.4.3. We also considered the posterior probabilities that were associated with each branch node when estimating timing of ancestry. Estimates of tMRCA were only generated for nodes with a posterior probability ≥0.90. Estimates of tMRCA for bifurcating nodes with lower support (<0.90) were not calculated, as these phylogenetic relationships were considered to be unresolved.

## RESULTS

### IAV detection and subtypes of isolates

From April 2014 through August 2015, we screened 3779 swab samples that had been collected in California from 2638 individual animals (1496 waterfowl and 1142 pinnipeds) for IAVs (Table 1). Samples originated from three sampling periods that were defined by our sampling methodology and related to the previously described outbreak of HP clade 2.3.4.4 H5 IAV in the Pacific Americas Flyway: pre-outbreak (April–August 2014); outbreak (September 2014–March 2015); and post-outbreak (April–August 2015).

IAV RNA was detected in 157 waterfowl samples (10.5% of birds sampled; 157/1496) and two swab samples that had been collected from pinnipeds (0.2% of pinnipeds sampled; 2/1142; Table 1). HP clade 2.3.4.4 H5 IAV RNA was identified in three cloacal swab samples (0.2% of birds sampled; 3/1496) collected in Butte (*n*=2) and Solano (*n*=1) counties originating from hunter-harvested American wigeon (*Anas americana*) sampled on 17 January 2015 (Table 1). When only considering samples that were collected from 20 December 2014 to 1 February 2015, the same time period that was used in a previous summary of surveillance efforts for HP clade 2.3.4.4 IAVs in the Pacific Americas Flyway,^16^ we detected RNA for clade 2.3.4.4 IAVs in 3/374 samples (0.8%) collected from hunter-harvested waterfowl (data not shown). Whole-genome sequencing was successfully performed on RNA that had been extracted from a single diagnostic sample, and was verified as belonging to a HP clade 2.3.4.4 IAV of the H5N8 subtype (strain name: A/American wigeon/California/UCD58P/2015(H5N8)).

A total of 86 LP IAV isolates were recovered from waterfowl that had been sampled throughout the entire April 2014–August 2015 sampling period, while none were isolated from either nasal or rectal swab samples collected from pinnipeds. From the 86 LP IAV isolates that originated from waterfowl, we amplified and genetically sequenced 57 partial or complete IAV genomes. Sequencing indicated that the three isolates for which genetic data were obtained represented mixed infections. The combined subtypes of 51 LP IAVs for which we obtained full genomes and for which we did not detect evidence of mixed infections included H1N1 (*n*=7), H2N5 (*n*=1), H2N6 (*n*=1), H3N8 (*n*=5), H4N6 (*n*=10), H4N8 (*n*=3), H5N2 (*n*=3), H6N1 (*n*=1), H7N3 (*n*=2), H7N4 (*n*=1), H8N4 (*n*=1), H10N2 (*n*=1) and H11N9 (*n*=15). The predominate combined subtype of LP IAV genomes from isolates recovered from waterfowl samples collected during the pre-outbreak period was H1N1 (6/9 or 67%), during the outbreak period was H5N2 (3/10 or 30%) and during the post-outbreak period was H11N9 (15/32 or 47%).

### Phylogenetic analyses

**Figure 2:**
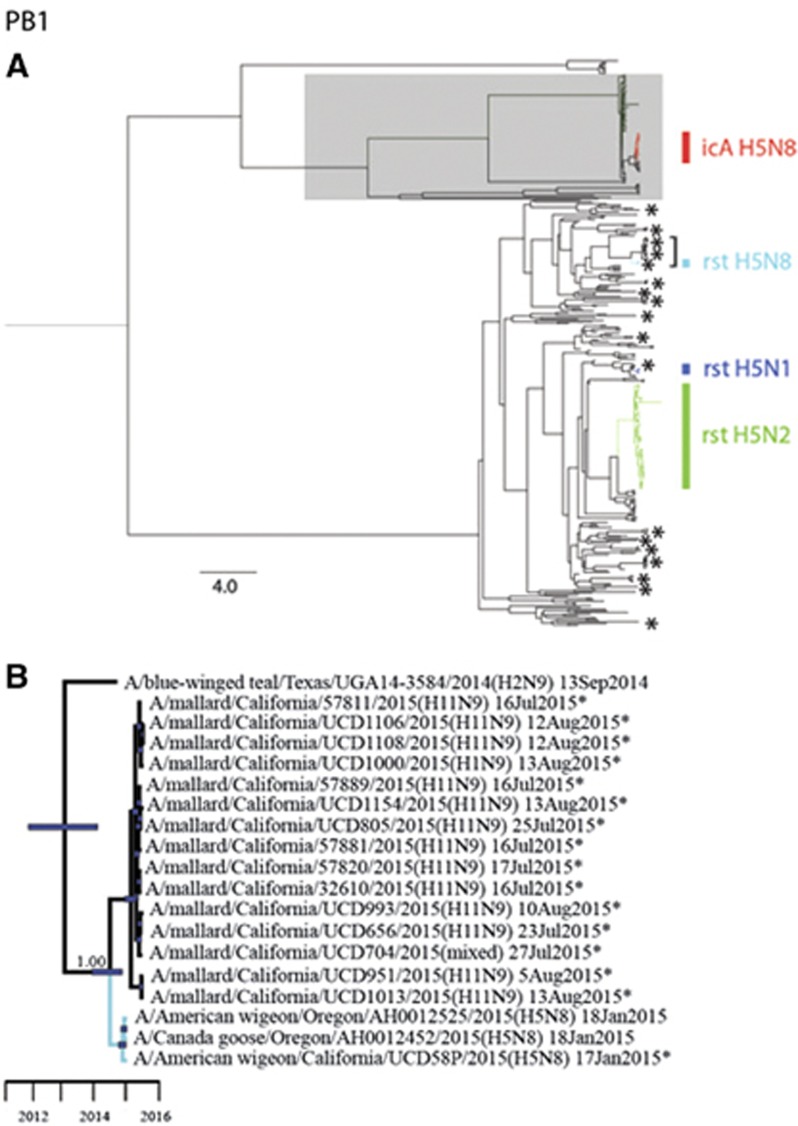
Bayesian phylogenetic tree depicting the inferred genetic relationship among PB1 gene segment sequences for isolates that originated from wild waterfowl in California during the period from 2014 to 2015, highly pathogenic (HP) clade 2.3.4.4 H5 influenza A viruses that were detected in North America during the concurrent outbreak and other reference sequences for wild bird low-pathogenic (LP) influenza A viruses that had been isolated from North America and East Asia. (**A**) The complete Bayesian phylogeny. Eurasian viral lineages are highlighted in gray. The approximate location of sequences of influenza A virus isolates from wild waterfowl that were sampled in California during 2014–2015 are depicted with asterisks. Asterisks represent more than one closely related sequence in some instances. Branch tips for clade 2.3.4.4 HP H5 influenza A viruses detected in North America are colored-coded and identified as follows: red=intercontinental lineage A HP H5N8 first introduced into North America; light blue=reassortant HP H5N8; dark blue=reassortant HP H5N1; and green=reassortant HP H5N2. The bracket indicates the portion of the phylogeny that is presented in panel B. Inferred evolutionary distance is indicated by the scale bar. (**B**) A partial PB1 phylogeny to depict inferred genetic ancestry among strain A/American wigeon/California/UCD58P/2015(H5N8), other reassortant HP H5N8 viruses that have been detected in North America and other LP influenza A viruses that were isolated in California as part of this study or elsewhere as part of concurrent surveillance efforts. Color is used to differentiate branch tips for HP reassortant H5N8 viruses (light blue) and LP viruses (black). Asterisks are used to indicate genetic sequences that were produced as part of the current study. Blue bars at nodes depict 95% highest posterior densities, which can be interpreted relative to the provided timescale. The posterior probability for the node depicting inferred common ancestry between reassortant HP H5N8 influenza A viruses and LP viruses detected in mallards sampled in California as part of this study is shown. Refer to Supplementary Figure S2 for the complete phylogenetic tree with tip labels.

**Figure 3:**
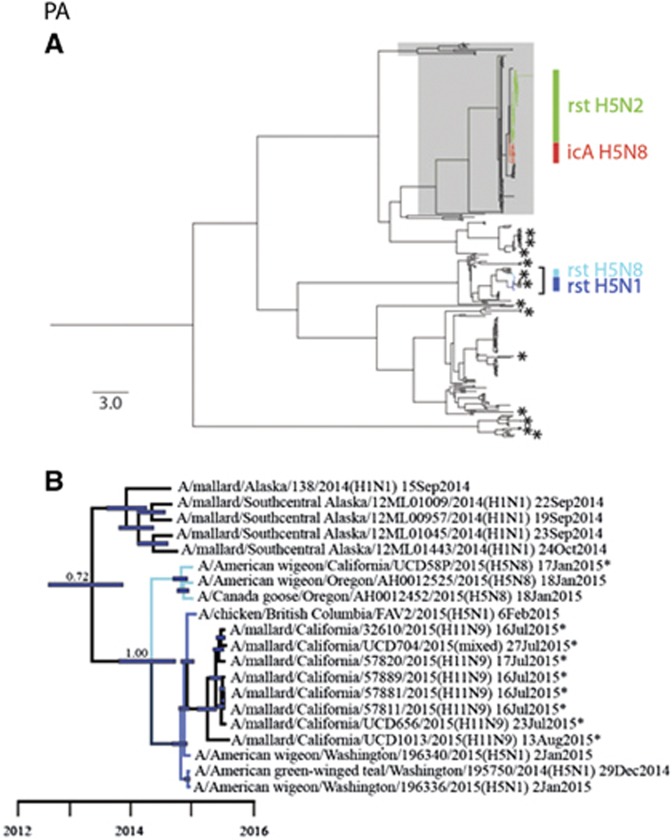
Bayesian phylogenetic trees depicting the inferred genetic relationship among PA gene segment sequences for isolates originating from wild waterfowl in California from 2014–2015, highly pathogenic (HP) clade 2.3.4.4 H5 influenza A viruses detected in North America during the concurrent outbreak and other reference sequences for wild bird low-pathogenic (LP) influenza A viruses isolated from North America and East Asia. (**A**) The complete Bayesian phylogeny. Eurasian viral lineages are highlighted in gray. The approximate location of sequences for influenza A virus isolates from wild waterfowl that were sampled in California during the period from 2014 to 2015 are depicted with asterisks. Asterisks represent more than one closely related sequence in some instances. Branch tips for clade 2.3.4.4 HP H5 influenza A viruses detected in North America are color-coded and identified as follows: red=intercontinental lineage A HP H5N8 that was first introduced into North America; light blue=reassortant HP H5N8; dark blue=reassortant HP H5N1; and green=reassortant HP H5N2. The bracket indicates the portion of the phylogeny that is presented in panel B. Inferred evolutionary distance is indicated by the scale bar. (**B**) partial PA phylogeny to depict inferred genetic ancestry among strain A/American wigeon/California/UCD58P/2015(H5N8), other reassortant HP H5N1 and H5N8 viruses detected in North America and other LP influenza A viruses that were isolated in California as part of this study or elsewhere as part of concurrent surveillance efforts. Color is used to differentiate among branch tips for HP reassortant H5N1 (dark blue), HP reassortant H5N8 (light blue) and LP influenza A viruses (black). Asterisks are used to indicate genetic sequences produced as part of the current study. Blue bars at nodes depict 95% highest posterior densities, which can be interpreted relative to the provided timescale. The posterior probability of a node that depicts inferred common ancestry between reassortant HP H5N1 and HP H5N8 influenza A viruses with LP viruses detected in mallards sampled in California as part of this study is shown. Refer to Supplementary Figure S3 for the complete phylogenetic tree with tip labels.

Phylogenetic analyses revealed that sequences for all eight gene segments of strain A/American wigeon/California/UCD58P/2015(H5N8) were clustered in monophyletic clades with other HP clade 2.3.4.4 IAVs that had been detected in western North America during 2014–2015 (Figures 2 and 3; Supplementary Figures S1–S8). However, PB1 and PA gene segment sequences for A/American wigeon/California/UCD58P/2015(H5N8) were both of North American lineages, in contrast to other HP clade 2.3.4.4 H5N8 IAVs that had been previously reported from California, A/chicken/California/15-004912/2015(H5N8) and A/turkey/California/K1500169-1.2/2015(H5N8), which were composed of completely Eurasian origin gene segments (Figures 2 and 3; Supplementary Figures S1–S8). The PB1 gene segment sequences for strains A/American wigeon/California/UCD58P/2015(H5N8), A/American wigeon/Oregon/AH0012525/2015(H5N8) and A/Canada goose/Oregon/AH0012452/2015(H5N8) formed a monophyletic clade that shared recent common ancestry with 14 H11Nx LP IAVs (including a mixed infection) and a single H1N9 LP IAV that circulated among mallards (*Anas platyrhynchos*) that had been sampled in California as part of this study (posterior probability=1.00; tMRCA: 13 June 2014, 95% HPD: 28 October 2013–14 November 2014; Figure 2; Supplementary Figure S2). Similarly, these three reassortant HP clade 2.3.4.4 H5N8 IAVs formed a monophyletic clade in our phylogeny for the PA gene segment, sharing common ancestry with four reassortant HP clade 2.3.4.4 H5N1 IAVs that had been detected in British Columbia, Canada and Washington, USA, as well as eight H11Nx LP IAVs (including a mixed infection) that had been detected in mallards sampled in California for this study (posterior probability=1.00; tMRCA: 25 May 2014, 95% HPD: 22 November 2013–8 October 2014; Figure 3; Supplementary Figure S3). This strongly supported clade of PA gene segment sequences also appeared to share ancestry with sequences for LP H1N1 IAVs that had been detected in mallards in Alaska during autumn 2014; however, a Bayesian posterior probability (0.72) that was lower than our *a priori* threshold precluded us from inferring that this relationship was resolved with confidence and thus estimating the tMRCA (Figure 3).

**Figure 4:**
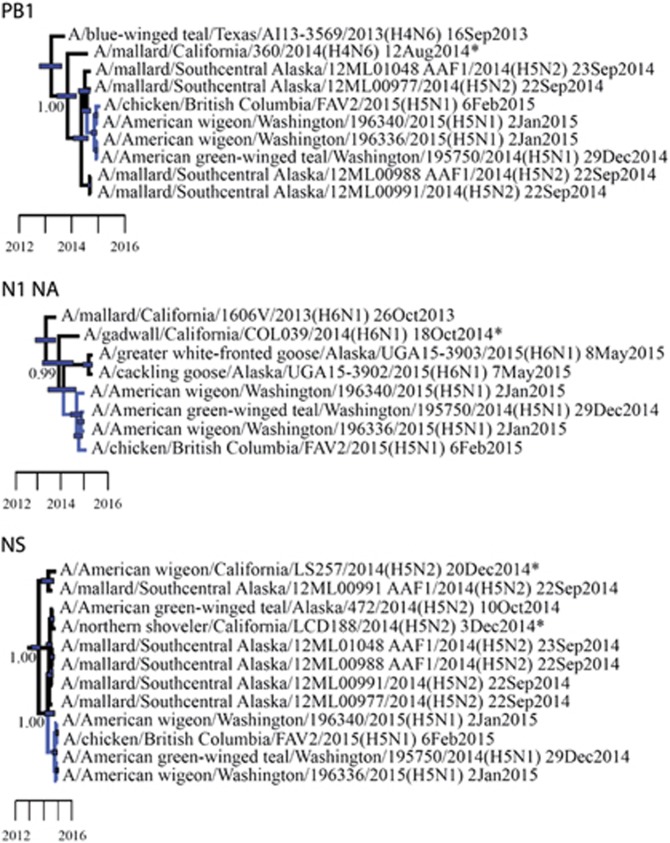
Partial Bayesian phylogenetic trees depicting the inferred genetic relationship among PB1, N1 NA and NS gene segment sequences for isolates that originated from wild waterfowl in California from 2014 to 2015 (indicated with asterisks), highly pathogenic (HP) clade 2.3.4.4 H5N1 influenza A viruses detected in North America during the concurrent outbreak and other reference sequences for wild bird low-pathogenic (LP) influenza A viruses isolated from elsewhere in North America as part of previous or concurrent surveillance efforts. Blue bars at nodes depict 95% highest posterior densities, which can be interpreted relative to the provided timescale. The posterior probability for nodes that depict inferred common ancestry among reassortant HP H5N1 influenza A viruses (dark blue branch tips) and LP viruses (black branch tips) detected in wild birds sampled in California as part of this study and previously in Alaska is shown. Refer to Supplementary Figures S2 (PB1), S8 (NS) and S9 (N1) for the complete phylogenetic trees with tip labels.

Numerous other gene segments for LP IAVs that were recovered from waterfowl samples collected in California during 2014–2015 shared relatively recent common ancestry with HP clade 2.3.4.4 reassortant viruses previously reported in North America. The *PB1* gene of A/mallard/California/360/2014(H4N6) shared relatively recent common ancestry with viruses that had been recovered in Alaska in September 2014 and reassortant HP clade 2.3.4.4 H5N1 IAVs recovered in British Columbia, Canada and Washington, USA in December 2014–February 2015 (posterior probability=1.00; tMRCA: 22 October 2013, 95% HPD: 23 May 2013–27 February 2014; Figure 4; Supplementary Figure S2). Similarly, strain A/gadwall/California/COL039/2014 (H6N1) shared recent common ancestry at the N1 NA gene segment with other H6N1 LP IAVs that had been detected in Alaska in 2015 and HP clade 2.3.4.4 reassortant H5N1 IAVs isolated in British Columbia, Canada, and Washington, USA, during the 2014–2015 outbreak (posterior probability=0.99; tMRCA: 5 December 2013, 95% HPD: 26 March 2013–13 July 2014; Figure 4; Supplementary Figure S9). NS gene segment sequences for two isolates recovered from an American wigeon (A/American wigeon/California/LS257/2014(H5N2)) and a northern shoveler (*Anas clypeata*; A/northern shoveler/California/LDC188/2014(H5N2)) also clustered in strongly supported clades with LP H5N2 subtype IAVs that had been recovered from waterfowl sampled in Alaska that shared common ancestry with reassortant HP clade 2.3.4.4 H5N1 IAVs (Figure 4). The clade composed of A/American wigeon/California/LS257/2014(H5N2) and another isolate recovered from a mallard sampled in Alaska was estimated to share ancestry with reassortant HP clade 2.3.4.4 H5N1 IAVs in June 2013 (posterior probability=1.00; tMRCA: 19 June 2013, 95% HPD: 1 March 2013–16 July 2013), which is before the estimate of shared common ancestry between reassortant HP H5N1 IAVs and the clade composed of strain A/northern shoveler/California/LDC188/2014(H5N2) and five isolates recovered from waterfowl sampled in Alaska (posterior probability=1.00; tMRCA: 9 January 2014, 95% HPD: 8 February 2014–6 September 2014; Figure 4; Supplementary Figure S8). The isolate A/northern shoveler/California/LDC188/2014 (H5N2) also appeared to be genetically closely related at the N2 NA gene segment to HP clade 2.3.4.4 H5N2 IAVs isolated from wild and domestic birds throughout the Pacific Northwest and Central USA during the 2014–2015 outbreak; however, a relatively low Bayesian posterior probability (0.31) precluded us from inferring that this relationship was resolved with confidence and estimating the tMRCA (Supplementary Figure S10). Gene segment sequences for other IAVs that had been isolated from waterfowl as part of this study clustered with those for IAVs recovered from wild birds throughout the Pacific Americas Flyway and other areas of North America (Figures 2 and 3; Supplementary Figures S1–S10).

## DISCUSSION

In this study, we consistently detected LP IAVs in waterfowl sampled in California during each of three sampling periods during April 2014–August 2015 and found a relatively low apparent prevalence of HP H5 clade 2.3.4.4 IAVs in wild waterfowl sampled in California, identifying clade 2.3.4.4 IAV RNA in three samples that were collected from American wigeon on 17 January 2015. These results are consistent with previous findings, including (1) the detection of LP IAVs in waterfowl sampled in California during both breeding^34^ and non-breeding periods,^35, 36^ (2) the relatively low prevalence of HP H5 clade 2.3.4.4 viruses in waterfowl sampled in the Pacific Americas Flyway during the 2014–2015 outbreak (0.8% in this study during 20 December 2014–1 February 2015 compared with 1.3% reported by Bevins *et al*,^16^) (3) the approximate seasonal timing at which HP H5 clade 2.3.4.4 IAVs were first and most frequently detected in California (first detected during the non-breeding season in 2014–2015, specifically 28 December 2014, and most commonly detected 10–17 January 2017)^16^ and (4) the most common host (American wigeon) among hunter-harvested waterfowl in which HP IAVs were identified during 20 December 2014–1 February 2015.^16^ Thus, our evaluation of the prevalence of HP clade 2.3.4.4 IAVs in waterfowl relative to previous reports supports prior conclusions regarding the proportion of sampled waterfowl hosts that were infected during the outbreak and the likely timing of introduction into California. With regard to the latter relationship, we acknowledge that our interpretation is based on limited sampling of waterfowl in California during the pre-outbreak period.

Pinnipeds that stranded at coastal areas of North America have previously been found to be positive for avian-origin IAVs.^20, 21, 22^ However, despite sampling a large number of animals that stranded along the Central Coast of California during 2014–2015, we found limited evidence of exposure of pinnipeds to LP IAVs and no evidence for infection with HP H5 clade 2.3.4.4 IAVs. It is unclear whether this result reflects a lack of exposure of marine mammals in California to HP IAVs during the 2014–2015 outbreak period, unidentified exposure biases that could have resulted from sampling stranded animals, poor infectivity of such viruses in pinnipeds or other unidentified factors. Alternatively, as we did not test for antibodies to HP clade 2.3.4.4 IAVs, it is plausible that California pinnipeds that were sampled as part of this study had been previously exposed to such viruses and that our sampling for active viral shedding was not sufficiently broad in time or space to detect evidence of infection. The development and application of a serologic test to assess marine mammal exposure to HP clade 2.3.4.4 IAVs would be helpful for determining whether pinnipeds had been exposed to such viruses.

As per our efforts to assess the spatiotemporal extent of viral lineages and to refine inference regarding evolutionary pathways of novel reassortant viruses by using genetic analyses, the HP single clade 2.3.4.4 virus for which we were able to generate complete genomic data was found to be of the H5N8 subtype. Again, this finding is consistent with a lack of detection of reassortant HP clade 2.3.4.4 H5N1 or H5N2 IAVs in wild or domestic birds in California during the 2014–2015 outbreak.^16^ Thus, it is plausible that reassortant HP clade 2.3.4.4 H5N1 and H5N2 viruses emerged at areas north of California and were never introduced to this state. Alternatively, the introduction of these reassortant viruses may have occurred, but this study and other surveillance efforts in California during 2014–2015 lacked sufficient sensitivity to detect such viruses in wild or domestic birds.

The HP clade 2.3.4.4 H5N8 IAV that was detected in an American wigeon and sequenced as part of this study is a reassortant virus, as compared to the icA HP H5N8 IAV that had been initially introduced into North America, with PB1 and PA gene segments closely related to those circulating in waterfowl sampled in California and elsewhere in the Pacific Americas Flyway. This result contrasts with previous reports of HP clade 2.3.4.4 H5N8 IAVs (in California poultry) that were composed entirely of Eurasian lineage gene segments and provides evidence that at least two genomic constellations of HP clade 2.3.4.4 IAVs were circulating in California during 2014–2015. Furthermore, as genomic constellations similar to strain A/American wigeon/California/UCD58P/2015(H5N8) have only been identified in waterfowl inhabiting the Pacific Americas Flyway, it is plausible that this reassortant emerged and was maintained during the 2014–2015 outbreak in wild birds, independent of domestic poultry.

Phylogenetic clustering of the reassortant HP H5N8 virus that was sequenced as part of this study with other HP clade 2.3.4.4 IAVs detected in western North America during 2014–2015 is congruent with previously proposed pathways of viral introduction and evolution within the Pacific Americas Flyway.^5, 11, 18, 19, 37^ In addition, estimates of relatively recent common ancestry of numerous gene segments (i.e., PB1, PA, N1 and NS) of reassortant HP clade 2.3.4.4 H5N1 and H5N8 viruses with LP IAVs from waterfowl sampled in California as part of this study further support the hypothesis that viruses that circulated in wild birds in the Pacific Americas Flyway were involved in reassortment events that led to the emergence of novel reassortant HP IAV genome constellations. This finding, similar to information that was previously reported in research on IAVs in waterfowl that had been sampled in Alaska during 2014–2015,^18, 19^ adds to the evidence that wild birds are likely to have played a role in the emergence and spread of novel reassortant HP clade 2.3.4.4 IAVs in North America.

**Figure 5:**
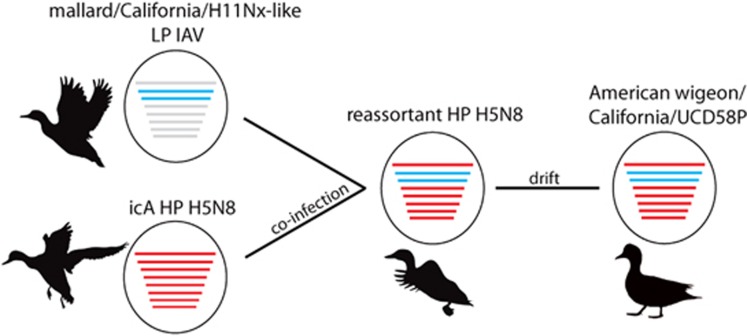
Proposed evolutionary pathway for influenza A virus strain A/American wigeon/California/UCD58P/2015(H5N8) sequenced as part of this study. Lines representing evolutionary mechanisms are labeled. Silhouettes depict that wild waterfowl were probable hosts for viruses along the proposed evolutionary pathway. The notation ‘mallard/California/H11Nx-like’ refers to an unsampled viral progenitor to H11Nx influenza A viruses that was detected in mallards sampled in California as part of this study sharing common ancestry at the PB1 and PA gene segments with reassortant HP H5N8 viruses. The subtype, host species and location for this progenitor virus are unknown. highly pathogenic, HP; influenza A virus, IAV; intercontinental lineage A clade 2.3.4.4 H5N8, icA; low pathogenic, LP.

On the basis of the results of our phylogenetic analyses, we propose a plausible evolutionary pathway for the HP clade 2.3.4.4 IAV strain A/American wigeon/California/UCD58P/2015(H5N8), which was detected in Solano County, California, as part of this study (Figure 5). The phylogenetic position of sequences for six gene segments (PB2, HA, NP, N8, and NS and M) of A/American wigeon/California/UCD58P/2015(H5N8) in Eurasian viral lineages and within monophyletic clades comprised other HP clade 2.3.4.4 IAVs that had been previously detected in wild and domestic birds in North America during the 2014–2015 outbreak, suggests that this virus most likely followed an evolutionary trajectory that involved reassortment between the HP icA H5N8 genome constellation that had been introduced into North America and other LP IAVs that circulated in wild waterfowl in the Pacific Americas Flyway. Given the relatively high posterior probability for shared ancestry at both the PB1 and the PA gene segments among three reassortant HP H5N8 IAVs that were detected in California and Oregon, and eight H11Nx LP IAVs that circulated in mallards that were sampled in California during 2015, as well as the numerous detections of the Eurasian icA HP H5N8 IAVs in wild waterfowl inhabiting the Pacific Americas Flyway in 2014–2015, it is probable that the genome constellation that ultimately led to the A/American wigeon/California/UCD58P/2015(H5N8) isolate was formed through a single reassortment event in a wild bird host. Furthermore, given (i) the timing and location of the first detection of clade 2.3.4.4 IAVs in North America, (ii) our estimates for the tMRCA at both the PB1 and the PA gene segments, (iii) the support for shared common ancestry between reassortant HP clade 2.3.4.4 H5N1 and HP H5N8 lineages at the PA gene segment and a lack of detection of reassortant HP H5N1 IAVs in California through surveillance efforts conducted to date and (iv) the prior detection of reassortant HP H5N1 and H5N8 lineages in areas north of California, it is likely that the reassortment event(s) that led to the emergence of this reassortant HP H5N8 genome constellation occurred north of California, during the breeding season or early migratory period for wild waterfowl, and within the Pacific Americas Flyway. While this evolutionary trajectory is supported through analyses in the current investigation, we acknowledge that numerous alternative pathways of viral evolution are also plausible.

Our phylogenetic analysis also provides support for relatively recent common ancestry at the PB1, PA, N1 NA and NS gene segments among LP IAVs that were recovered from waterfowl sampled in California during 2014–2015 and reassortant HP clade 2.3.4.4 H5N1 IAVs detected in British Columbia, Canada, and Washington, USA. Viral lineages that were detected in LP IAVs in waterfowl sampled in California for these four gene segments (PB1, PA, N1 NA and NS) therefore broadly represent all the North American lineage viral contributions to the clade 2.3.4.4 HP H5N1 genome constellation detected in the Pacific Americas Flyway during 2014–2015. Given that closely related PB1, PA, N1 NA and NS gene segment sequences were also detected in isolates derived from wild birds sampled in Alaska during 2014–2015, it is likely that such genetic diversity was widespread throughout the Pacific Americas Flyway at the time that novel reassortant HP clade 2.3.4.4 H5N1 viruses first emerged. Furthermore, as no IAV isolate from California sequenced as part of this study contained more than a single gene segment that shared recent common ancestry with clade 2.3.4.4 HP H5N1 IAVs, which is comparable to data for LP IAVs from Alaska in which no more than two gene segments that shared recent common ancestry with a reassortant HP H5N1 IAV were identified in any given isolate,^19^ either multiple reassortment events led to the emergence of the HP clade 2.3.4.4 H5N1 genome constellation in North America or the genome constellation that served as a predecessor to this virus, via reassortment with the icA HP H5N8 initially introduced into North America, has not yet been identified. Thus, while this study provides important new data on genetic diversity for IAVs that were circulating among waterfowl in California during 2014–2015 and complements previously published data from Alaska, additional genetic information for wild waterfowl-origin LP IAVs from other locations in the Pacific Flyway during this time period would be useful for estimating the time and place of the emergence of this reassortant HP clade 2.3.4.4 H5N1 IAV genome constellation.

While we did not detect HP clade 2.3.4.4 H5 IAVs in wild waterfowl sampled in California in the summer of 2015 despite sampling IAV-naive hatch-year birds at the same location where HP H5N8 had been identified 6 months prior, we caution against drawing the conclusion that such viruses have disappeared from this region or elsewhere in North America. Although it has been suggested that HP IAVs are adapted to domestic birds and unable to be maintained in wild waterfowl,^38^ recent detection of HP clade 2.3.4.4 H5N2 IAVs in wild mallards in Alaska^39^ and Montana,^40^ (which have seemingly been absent from domestic birds in North America since mid-2015), widespread detection of HP H5Nx IAVs in wild birds in Europe, Africa and Asia from 2016 to 2017,^41^ and pathobiological data from laboratory experiments that support the adaption of HP clade 2.3.4.4 H5 IAVs to waterfowl,^17, 42, 43^ collectively provide evidence that this conceptual model may be inaccurate for numerous contemporary viral lineages descended from HP H5 Goose Guangdong lineage IAV predecessors.^44^ While information provided in this study and a previous report by Bevins *et al.*^16^ provide data regarding the circulation of clade 2.3.4.4 IAVs in waterfowl in the Pacific Americas Flyway, we do not know the encounter and detection probabilities for such viruses in surveillance efforts, how prevalence varied across space and time during and after the 2014–2015 outbreak and how environmental persistence may contribute to viral ecology of clade 2.3.4.4 IAVs in North America. Thus, continued surveillance for HP IAVs in California and elsewhere in North America is warranted, as is vigilance with regard to adhering to biosecurity recommendations to limit the contact of domestic birds with water, soil or fomites that may have come into contact with wild waterfowl or wild bird feces.

## Figures and Tables

**Table 1:** Summary of surveillance sampling of wild waterfowl and pinnipeds in California for HP clade 2.3.4.4 H5 influenza A viruses in wild waterfowl during 2014–2015

	**Pre-outbreak (April–August 2014)**					**Outbreak (September 2014–March 2015)**					**Post-outbreak (April–August 2015)**				
**Species**	***n***	**MA+**^a^ **(%)**	**icA+**^b^	**VI+**^c^	**VI+/MA+ (%)**	***n***	**MA+ (%)**	**icA+**	**VI+**	**VI+/MA+ (%)**	***n***	**MA+ (%)**	**icA+**	**VI+**	**VI+/MA+ (%)**
*Waterfowl*^d^															
American green-winged teal (*Anas crecca*)	NT	NT	NT	NT	NT	171	7 (4%)	0	0	0/7 (0%)	1	0 (0%)	0	0	NT
American wigeon (*Anas americana*)	NT	NT	NT	NT	NT	185	8 (4%)	3	3	3/5 (60%)^e^	NT	NT	NT	NT	NT
Bufflehead (*Bucephala albeola*)	NT	NT	NT	NT	NT	29	1 (3%)	0	1	1/1 (100%)	NT	NT	NT	NT	NT
Common goldeneye (*Bucephala clangula*)	NT	NT	NT	NT	NT	11	1 (9%)	0	0	0/1 (0%)	NT	NT	NT	NT	NT
Gadwall (*Anas strepera*)	NT	NT	NT	NT	NT	57	2 (4%)	0	1	1/2 (50%)	41	0 (0%)	0	0	NT
Mallard (*Anas platyrhynchos*)															
Cloacal swab only	61	13 (21%)	0	9	9/12 (75%)^f^	76	6 (8%)	0	1	1/6 (17%)	487	81 (17%)	0	55	55/80 (69%)^f^
Combined cloacal/oropharyngeal swab	NT	NT	NT	NT	NT	NT	NT	NT	NT	NT	128	25 (20%)	0	8	8/22 (36%)^f^
Northern pintail (*Anas acuta*)	NT	NT	NT	NT	NT	52	0 (0%)	0	0	NT	1	1 (100%)	0	0	0 (0%)
Northern shoveler (*Anas clypeata*)	NT	NT	NT	NT	NT	99	11 (11%)	0	7	7/11 (64%)	NT	NT	NT	NT	NT
Ruddy duck (*Oxyura jamaicensis*)	NT	NT	NT	NT	NT	9	1 (11%)	0	1	1/1 (100%)	NT	NT	NT	NT	NT
*Marine mammals*^g^															
California sea lion (*Zalophus californianus*)															
Nasal swabs	345	0 (0%)	NT	0	NT	353	1 (<1%)	0	0	0/1 (0%)	81	0 (0%)	0	0	NT
Rectal swabs	345	0 (0%)	NT	0	NT	352	0 (0%)	0	0	NT	81	0 (0%)	0	0	NT
Pacific harbor seal (*Phoca vitulina richardii*)															
Nasal swabs	36	0 (0%)	NT	0	NT	29	0 (0%)	0	0	NT	68	0 (0%)	0	0	NT
Rectal swabs	36	0 (0%)	NT	0	NT	29	0 (0%)	0	0	NT	68	1 (1%)	0	0	0 (0%)

Abbreviations: highly pathogenic, HP; clade 2.3.4.4 HP H5 or intercontinental lineage A positive samples, icA+ matrix-positive samples, MA+ not tested, NT; number of samples collected, *n*; virus isolation positive samples, VI+.

All waterfowl samples are swabs collected from the cloaca only unless otherwise specified.

a MA+ defined as Ct value ≤45 using real-time reverse transcriptase-PCR (rRT-PCR) for matrix gene target (Runstadler *et al.*^26^).

b icA+ defined as having Ct value <40 using rRT-PCR for clade 2.3.4.4 hemagglutinin gene target (see Materials and Methods).

c VI+ defined as collected allantoic fluid with Ct value ≤45 using rRT-PCR for matrix gene target (Runstadler *et al.*^26^).

d Negative rRT-PCR results targeting matrix gene product were not included in the table for the following species: American coot (*Fulica americana*; *n*=2); Blue-winged teal (*Anas discors*; *n*=1); Cackling goose (*Branta hutchinsii*; *n*=1); Canada goose (*Branta canadensis*; cloacal swab *n*=1; fecal swab *n*=3); Cinnamon teal (*Anas cyanoptera*; *n*=6); Gadwall (*Anas strepera*; combined cloacal/oropharyngeal swab *n*=1); Greater white-fronted goose (*Anser albifrons*; *n*=15); Lesser scaup (*Aythya affinis*, *n*=3); Mallard (*Anas platyrhynchos*; oropharyngeal swab only *n*=2; fecal swab *n*=25); Ring-necked duck (*Aythya collaris*; *n*=11); Ross’s goose (*Chen rossii*; *n*=1); Snow goose (*Chen caerulescens*; *n*=5); and Wood duck (*Aix sponsa*; *n*=8).

e Diagnostic icA+ samples not subjected to VI at UC Davis; therefore, results were reported excluding icA+ samples.

f Not all samples were subjected to virus isolation on account of logistical considerations (e.g., insufficient sample volume).

g Negative rRT-PCR results targeting matrix gene product were not included in the table for paired nasal and rectal swab samples (processed separately) for the following species: Guadalupe fur seal (*Arctocephalus townsendi*; *n*=24); northern elephant seal (*Mirounga angustirostris*; *n*=175); northern fur seal (*Callorhinus ursinus*; *n*=30); and Steller sea lion (*Eumetopias jubatus*; *n*=1).

## References

[bib1] Li KS, Guan Y, Wang J et al. Genesis of a highly pathogenic and potentially pandemic H5N1 influenza virus in eastern Asia. Nature 2004; 430: 209–213. 15241415 10.1038/nature02746

[bib2] Zhao G, Gu X, Lu X et al. Novel reassortant highly pathogenic H5N2 avian influenza viruses in poultry in China. PLoS One 2012; 7: e46183. 23049973 10.1371/journal.pone.0046183 PMC3458027

[bib3] Zhao K, Gu M, Zhong L et al. Characterization of three H5N5 and one H5N8 highly pathogenic avian influenza viruses in China. Vet Microbiol 2013; 163: 351–357. 23375651 10.1016/j.vetmic.2012.12.025

[bib4] Qi X, Cui L, Yu H et al. Whole-genome sequence of a reassortant H5N6 avian influenza virus isolated from a live poultry market in China, 2013. Genome Announc 2014; 2: e00706–e00714. 25212611 10.1128/genomeA.00706-14 PMC4161740

[bib5] Pasick J, Berhane Y, Joseph T et al. Reassortant highly pathogenic influenza A H5N2 virus containing gene segments related to Eurasian H5N8 in British Columbia, Canada, 2014. Sci Rep 2015; 5: 9484. 25804829 10.1038/srep09484 PMC4372658

[bib6] Ip HS, Torchetti MK, Crespo R et al. Novel Eurasian highly pathogenic avian influenza A H5 viruses in wild birds, Washington, USA, 2014. Emerg Infect Dis 2015; 21: 886–890. 25898265 10.3201/eid2105.142020 PMC4412248

[bib7] Torchetti MK, Killian ML, Dusek RJ et al. Novel H5 clade 2.3. 4.4 reassortant (H5N1) virus from a green-winged teal in Washington, USA. Genome Announc 2015; 3: e00195-15. 25838478 10.1128/genomeA.00195-15 PMC4384482

[bib8] Verhagen JH, van der Jeugd HP, Nolet BA et al. Wild bird surveillance around outbreaks of highly pathogenic avian influenza A (H5N8) virus in the Netherlands, 2014, within the context of global flyways. Euro Surveill 2015; 20: 21069. 25846491 10.2807/1560-7917.es2015.20.12.21069

[bib9] Bi Y, Chen Q, Wang Q et al. Genesis, evolution and prevalence of H5N6 avian influenza viruses in China. Cell Host Microbe 2016; 20: 810–821. 27916476 10.1016/j.chom.2016.10.022

[bib10] Lee MS, Chen LH, Chen YP et al. Highly pathogenic avian influenza viruses H5N2, H5N3, and H5N8 in Taiwan in 2015. Vet Microbiol 2016; 187: 50–57. 27066708 10.1016/j.vetmic.2016.03.012

[bib11] Lee DH, Torchetti MK, Winker K et al. Intercontinental spread of Asian-origin H5N8 to North America through Beringia by migratory birds. J Virol 2015; 89: 6521–6524. 25855748 10.1128/JVI.00728-15 PMC4474297

[bib12] Miller MR, Takekawa JY, Fleskes JP et al. Spring migration of northern pintails from California's Central Valley wintering area tracked with satellite telemetry: routes, timing, and destinations. Can J Zool 2005; 83: 1314–1332.

[bib13] Hupp JW, Schmutz JA, Ely CR et al. Moult migration of emperor geese *Chen canagica* between Alaska and Russia. J Avian Biol 2007; 38: 462–470.

[bib14] Hupp JW, Yamaguchi N, Flint PL et al. Variation in spring migration routes and breeding distribution of northern pintails *Anas acuta* that winter in Japan. J Avian Biol 2011; 42: 289–300.

[bib15] Ramey AM, Reeves AB, Sonsthagen SA et al. Dispersal of H9N2 influenza A viruses between East Asia and North America by wild birds. Virology 2015; 482: 79–83. 25827532 10.1016/j.virol.2015.03.028

[bib16] Bevins SN, Dusek RJ, White CL et al. Widespread detection of highly pathogenic H5 influenza viruses in wild birds from the Pacific flyway of the United States. Sci Rep 2016; 6: 28980. 27381241 10.1038/srep28980 PMC4933915

[bib17] Pantin-Jackwood MJ, Costa-Hurtado M, Shepherd E et al. Pathogenicity and transmission of H5 and H7 highly pathogenic avian influenza viruses in mallards. J Virol 2016; 90: 9967–9982. 27558429 10.1128/JVI.01165-16 PMC5068544

[bib18] Ramey AM, Reeves AB, TeSlaa JL et al. Evidence for common ancestry among viruses isolated from wild birds in Beringia and highly pathogenic intercontinental reassortant H5N1 and H5N2 influenza A viruses. Infect Genet Evol 2016; 40: 176–185. 26944444 10.1016/j.meegid.2016.02.035

[bib19] Hill NJ, Hussein ITM, Davis KR et al. Reassortment of influenza A viruses in wild birds in Alaska before H5 clade 2.3.4.4 outbreaks. Emerg Infect Dis 2017; 23: 654–657. 28322698 10.3201/eid2304.161668 PMC5367406

[bib20] St Aubin DJ, Barker IK, Webster RG et al. Mass mortality of harbor seals: pneumonia associated with influenza A virus. Science 1982; 215: 1129–1131. 7063847 10.1126/science.7063847

[bib21] Callan RJ, Early G, Kida H et al. The appearance of H3 influenza viruses in seals. J Gen Virol 1995; 76: 199–203. 7844533 10.1099/0022-1317-76-1-199

[bib22] Anthony SJ, Leger JS, Pugliares K et al. Emergence of fatal avian influenza in New England harbor seals. MBio 2012; 3: e00166-12. 22851656 10.1128/mBio.00166-12 PMC3419516

[bib23] Goldstein T, Mena I, Anthony SJ et al. Pandemic H1N1 influenza isolated from free-ranging Northern Elephant Seals in 2010 off the central California coast. PLoS One 2013; 8: e62259. 23690933 10.1371/journal.pone.0062259 PMC3655164

[bib24] Boyce WM, Mena I, Yochem PK et al. Influenza A (H1N1) pdm09 virus infection in marine mammals in California. Emerg Microbes Infect 2013; 1: e40. 10.1038/emi.2013.40 PMC3698372 26038474

[bib25] Puryear WB, Keogh M, Hill N et al. Prevalence of influenza A virus in live-captured North Atlantic gray seals: a possible wild reservoir. Emerg Microbes Infect 2016; 5: e81. 27485496 10.1038/emi.2016.77 PMC5034098

[bib26] Runstadler JA, Happ GM, Slemons RD et al. Using RRT-PCR analysis and virus isolation to determine the prevalence of avian influenza virus infections in ducks at Minto Flats State Game Refuge, Alaska, during August 2005. Arch Virol 2007; 152: 1901–1910. 17541700 10.1007/s00705-007-0994-1 PMC2538573

[bib27] Mena I, Nelson MI, Quezada-Monroy F et al. Origins of the 2009 H1N1 influenza pandemic in swine in Mexico. eLIFE 2016; 5: e16777. 27350259 10.7554/eLife.16777 PMC4957980

[bib28] Kearse M, Moir R, Wilson A et al. Geneious Basic: an integrated and extendable desktop software platform for the organization and analysis of sequence data. Bioinformatics 2012; 28: 1647–1649. 22543367 10.1093/bioinformatics/bts199 PMC3371832

[bib29] Bao Y, Bolotov P, Dernovoy D et al. The influenza virus resource at the national center for biotechnology information. J Virol 2008; 82: 596–601. 17942553 10.1128/JVI.02005-07 PMC2224563

[bib30] Edgar RC. MUSCLE: multiple sequence alignment with high accuracy and high throughput. Nucleic Acids Res 2004; 32: 1792–1797. 15034147 10.1093/nar/gkh340 PMC390337

[bib31] Stamatakis AP. RAxML-VI-HPC: maximum likelihood-based phylogenetic analyses with thousands of taxa and mixed models. Bioinformatics 2006; 22: 2688–2690. 16928733 10.1093/bioinformatics/btl446

[bib32] Drummond AJ, Rambaut A. BEAST: Bayesian evolutionary analysis by sampling trees. BMC Evol Biol 2007; 7: 214. 17996036 10.1186/1471-2148-7-214 PMC2247476

[bib33] Drummond AJ, Suchard MA, Xie D et al. Bayesian phylogenetics with BEAUti and the BEAST 1.7. Mol Biol Evol 2012; 29: 1969–1973. 22367748 10.1093/molbev/mss075 PMC3408070

[bib34] Henaux V, Samuel MD, Dusek RJ et al. Presence of avian influenza viruses in waterfowl and wetlands during summer 2010 in California: are resident birds a potential reservoir? PLoS One 2012; 7: e31471. 22328934 10.1371/journal.pone.0031471 PMC3273456

[bib35] Pearce JM, Ramey AM, Flint PL et al. Avian influenza at both ends of a migratory flyway: characterizing viral genomic diversity to optimize surveillance plans for North America. Evol Appl 2009; 2: 457–468. 25567891 10.1111/j.1752-4571.2009.00071.x PMC3352445

[bib36] Hill NJ, Takekawa JY, Cardona CJ et al. Waterfowl ecology and avian influenza in California: do host traits inform us about viral occurrence? Avian Dis 2010; 54: 426–432. 20521673 10.1637/8912-043009-Reg.1

[bib37] Lee DH, Bahl J, Torchetti MK et al. Highly pathogenic avian influenza viruses and generation of novel reassortants, United States, 2014-2015. Emerg Infect Dis 2016; 22: 1283–1285. 27314845 10.3201/eid2207.160048 PMC4918163

[bib38] Krauss S, Stallknecht DE, Slemons RD et al. The enigma of the apparent disappearance of Eurasian highly pathogenic H5 clade 2.3. 4.4 influenza A viruses in North American waterfowl. Proc Natl Acad Sci USA 2016; 113: 9033–9038. 27457948 10.1073/pnas.1608853113 PMC4987810

[bib39] Lee D, Kim Torchetti M, Killian M et al. Reoccurrence of avian influenza A(H5N2) virus clade 2.3.4.4 in wild birds, Alaska, USA, 2016. Emerg Infect Dis 2017; 23: 365–367. 28098546 10.3201/eid2302.161616 PMC5324823

[bib40] US Department of Agriculture (USDA) Wild bird positive highly pathogenic avian influenza cases in the United States: July 2016 to June 2017. Available at https://www.aphis.usda.gov/animal_health/downloads/animal_diseases/ai/uspositivecases17.pdf (accessed 24 January 2017).

[bib41] Office International des Epizooties (OIE) Update on highly pathogenic avian influenza in animals (type H5 and H7). Available at http://www.oie.int/en/animal-health-in-the-world/update-on-avian-influenza/2017/ (accessed 24 January 2017).

[bib42] Kang HM, Lee EK, Song BM et al. Novel reassortant influenza A (H5N8) viruses among inoculated domestic and wild ducks, South Korea, 2014. Emerg Infect Dis 2015; 21: 298–304. 25625281 10.3201/eid2102.141268 PMC4313655

[bib43] DeJesus E, Costa-Hurtado M, Smith D et al. Changes in adaptation of H5N2 highly pathogenic avian influenza H5 clade 2.3. 4.4 viruses in chickens and mallards. Virology 2016; 499: 52–64. 27632565 10.1016/j.virol.2016.08.036 PMC5102764

[bib44] Ramey AM, Spackman E, Kim-Torchetti M et al. Weak support for disappearance and restricted emergence/persistence of highly pathogenic influenza A in North American waterfowl. Proc Natl Acad Sci USA 2016; 113: E6551–E6552. 27791026 10.1073/pnas.1614530113 PMC5087006

