# Supplementary figures and images for: Surveillance for highly pathogenic influenza A viruses in California during 2014–2015 provides insights into viral evolutionary pathways and the spatiotemporal extent of viruses in the Pacific Americas Flyway
Source: Emerg Microbes Infect. 2017 Sep 6;6(9):e80–. doi: 10.1038/emi.2017.66 (PMC5625317; doi:10.1038/emi.2017.66)

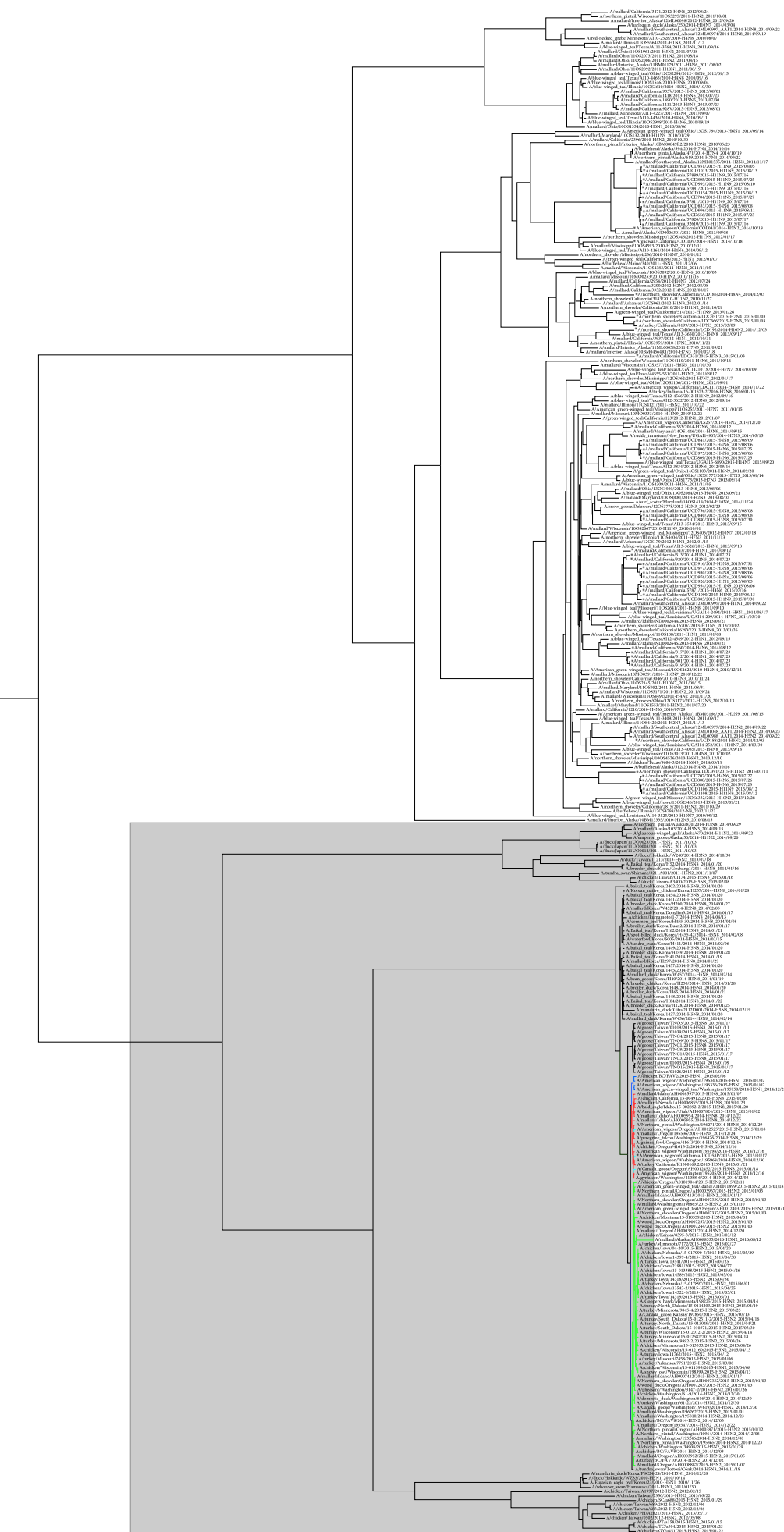

Supplement: Supplementary Figure S1 [file emi201766x1.pdf]

PB 1

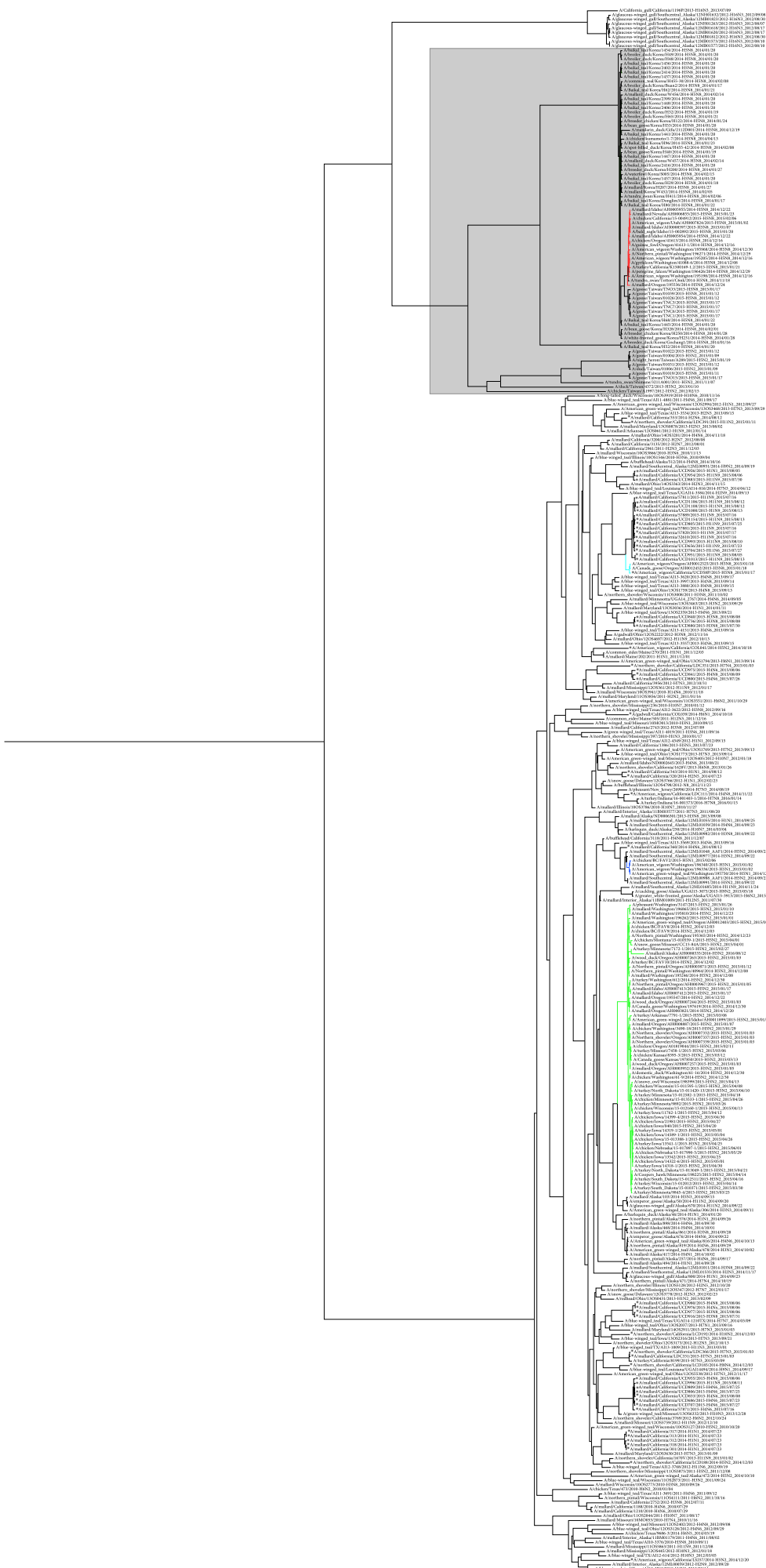

Supplement: Supplementary Figure S2 [file emi201766x2.pdf]

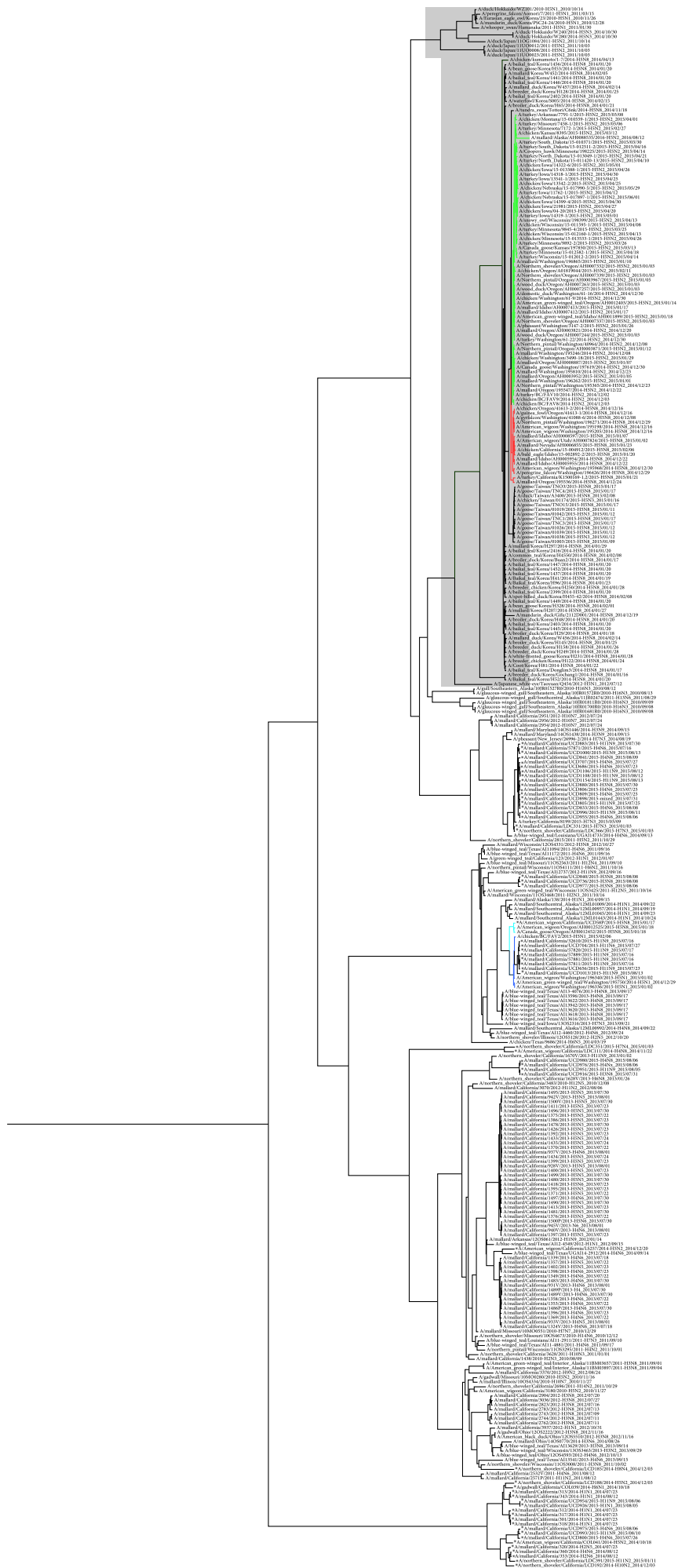

Supplement: Supplementary Figure S3 [file emi201766x3.pdf]

H5

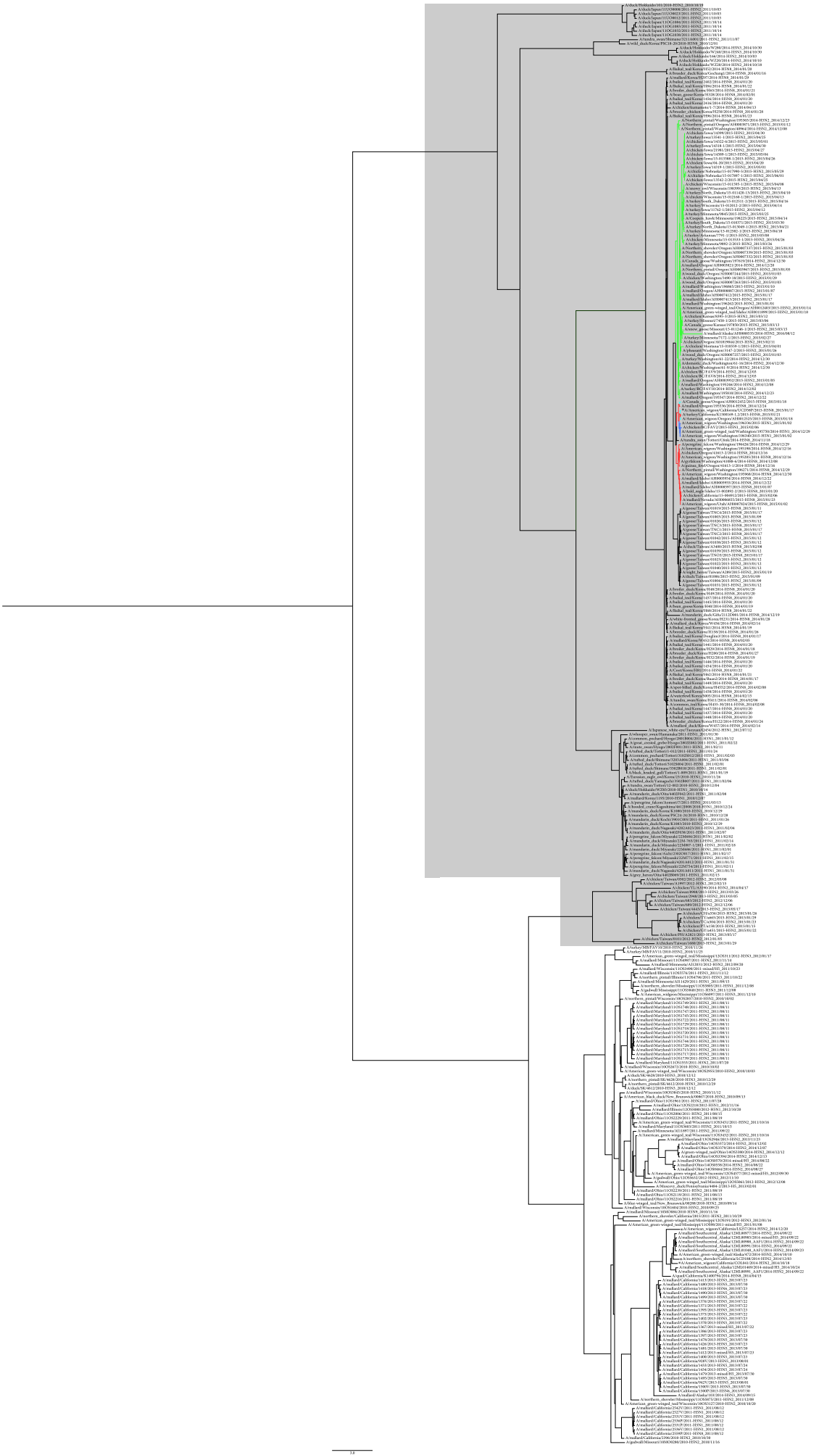

Supplement: Supplementary Figure S4 [file emi201766x4.pdf]

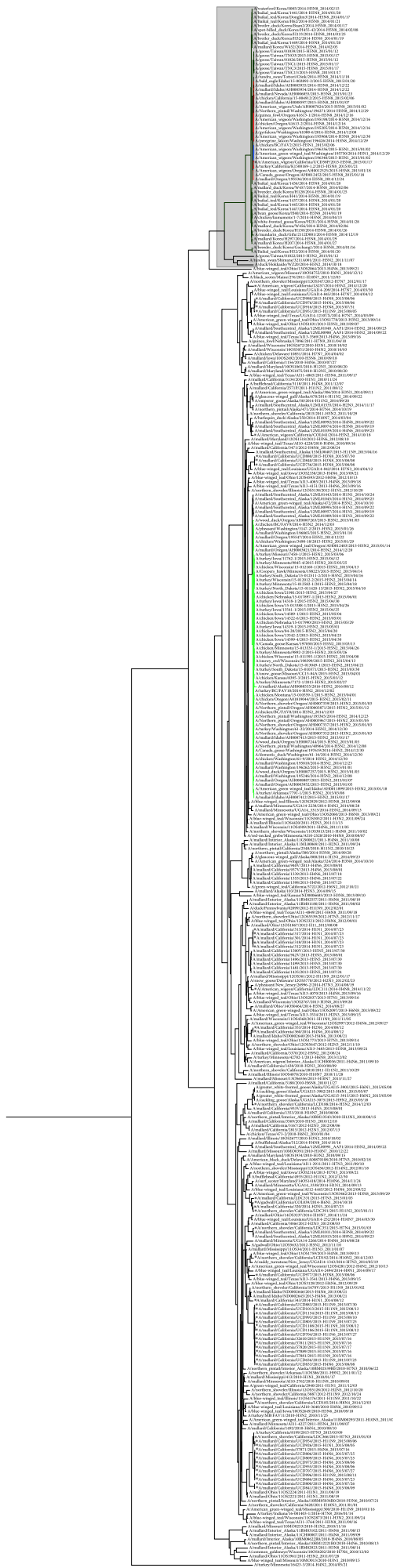

Supplement: Supplementary Figure S5 [file emi201766x5.pdf]

M

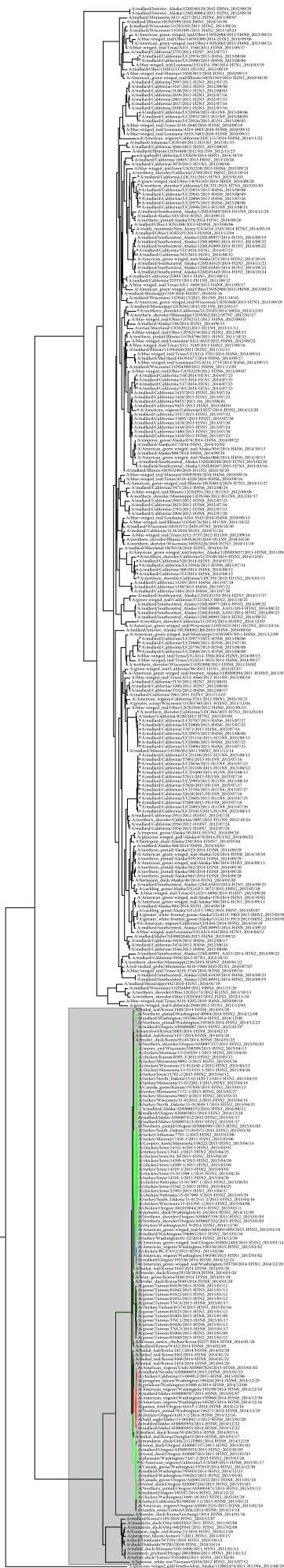

Supplement: Supplementary Figure S7 [file emi201766x7.pdf]

N1

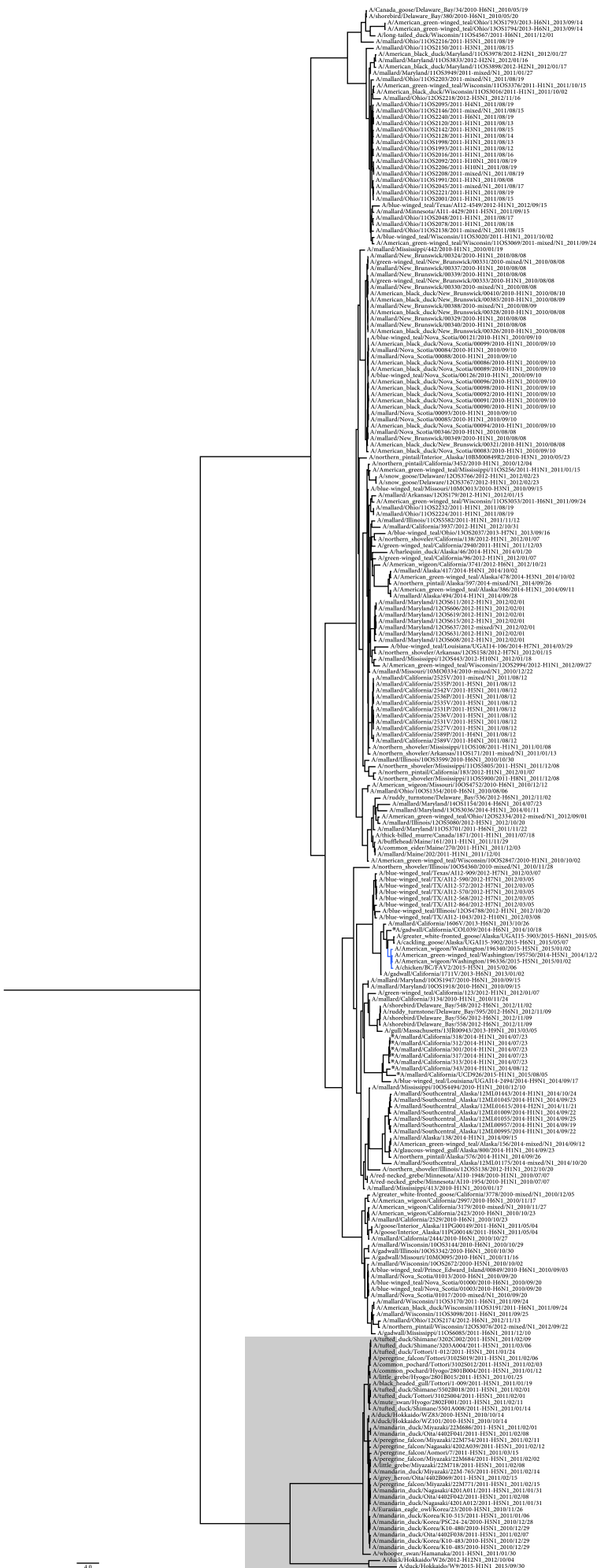

Supplement: Supplementary Figure S9 [file emi201766x9.pdf]
