# Supplementary material for: Surveillance for highly pathogenic influenza A viruses in California during 2014–2015 provides insights into viral evolutionary pathways and the spatiotemporal extent of viruses in the Pacific Americas Flyway
Source: Emerg Microbes Infect. 2017 Sep 6;6(9):e80–. doi: 10.1038/emi.2017.66 (PMC5625317; doi:10.1038/emi.2017.66)

# N2

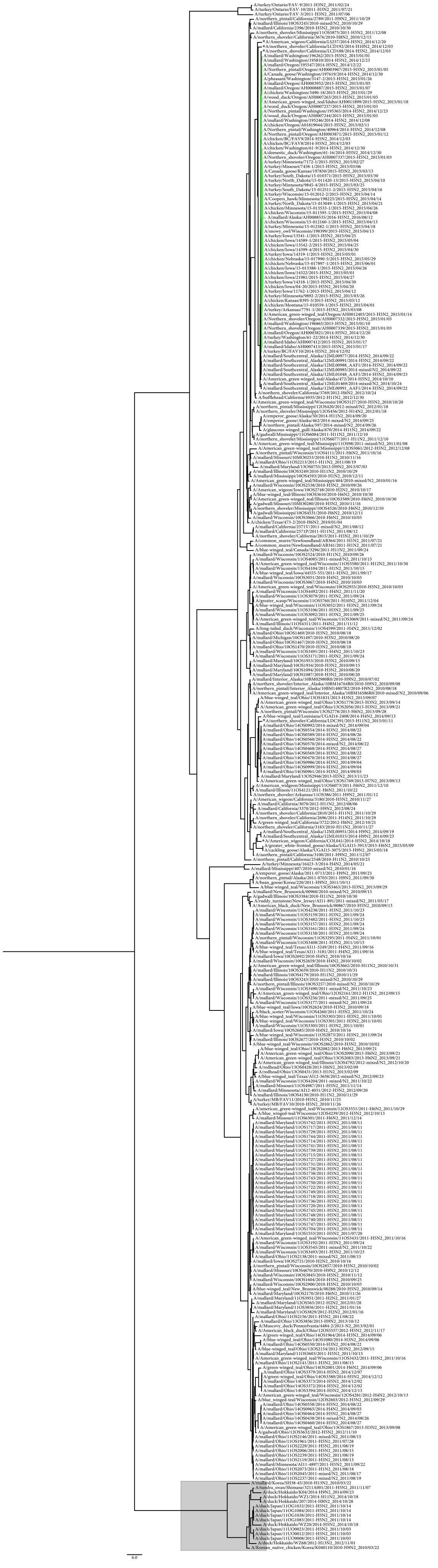

Supplement: Supplementary Figure S10 [file emi201766x10.pdf]
